# Men’s Knowledge, Attitudes, Practices, Cultural Beliefs, and Perceived Risk and Susceptibility Regarding Prostate Cancer in the Vhembe District, Limpopo Province, South Africa

**DOI:** 10.1007/s40615-025-02540-4

**Published:** 2025-07-17

**Authors:** Sean Mark Patrick, Joyce Shirinde, Vhuli Obida, Zazi Zikalala, Vanessa Hayes, Riana Bornman

**Affiliations:** 1 School of Health Systems and Public Health, University of Pretoria, Pretoria, South Africa; 2 Ancestry and Health Genomics Laboratory, Charles Perkins Centre, School of Medical Sciences, Faculty of Medicine and Health, University of Sydney, Camperdown, NSW 2050 Australia; 3 Manchester Cancer Research Centre, University of Manchester, Manchester, M20 4GJ UK

**Keywords:** Prostate Cancer, Men, KAP Survey, Cultural Beliefs, Vhembe District, South Africa

## Abstract

**Background:**

Prostate cancer (PCa) awareness and knowledge among men in Vhembe District, Limpopo Province, South Africa, remain inadequately studied despite the high local burden of the disease. This study investigates the knowledge, attitudes, practices, cultural beliefs, and perceived risk of PCa among men aged 40 and above in selected villages under the Mphaphuli and Niani tribal authorities.

**Methods:**

A quantitative survey was conducted with 431 men, utilizing a questionnaire adapted from the African Women Awareness of Cancer (AWACAN) tool. The questionnaire, translated into Tshivenda, assessed socio-demographic data, awareness, knowledge of risk factors and symptoms, health-seeking behavior, and barriers to seeking medical help.

**Results:**

The study revealed that 51.3% of participants had heard of PCa, while 48.7% had not. Awareness varied significantly with age, relationship status, education level, and language. Older men and those with higher education levels were more knowledgeable about PCa. Clinics, hospitals, and media were the primary sources of information. Misconceptions about risk factors were prevalent, with 24.0% of men indicating a preference for traditional healers for PCa symptoms. Barriers to medical help included fear of the disease, procedural fears, and cultural taboos. Multivariate analysis identified significant factors associated with PCa knowledge, including age, language, access to tap water, and cell phone ownership.

**Conclusion:**

These findings underscore the importance of targeted educational interventions considering sociodemographic and cultural contexts. Future public health initiatives should focus on bridging the gap between traditional and modern medical practices to enhance health outcomes in the Vhembe District and similar settings.

## Introduction

Prostate cancer (PCa) is one of the leading causes of cancer-related deaths among men globally. In 2022, there were 103,050 new cases of PCa, constituting 20.4% of all male cancer diagnoses, ranking PCa as having the fourth highest incidence rate among all cancers globally [1]. In Southern Africa, PCa was the most diagnosed cancer among males in 2022, accounting for 24.5% of all new male cancer cases, 5790 deaths, ranking it as the third major cause of cancer death in males [1]. Prostate cancer incidence and mortality rates vary widely due to detection practices, treatment availability, and genetics [2, 3]. Recent trends show stabilization or decline in many countries, driven by reduced PSA testing and improved treatments, especially in high-income countries [4]. While PCa cases are projected to double by 2040, research mainly focuses on men of European heritage, despite significant ethnic differences [5].

In Africa, high PCa mortality rates are exacerbated by late diagnoses and limited access to advanced treatments [6]. Urgent improvements in healthcare infrastructure, education, and early detection are needed to address the rising burden and prevent high mortality rates [4]. These interventions should include education, outreach, shifting to early-stage diagnosis, and curative treatments [5]. However, research disparities highlight the need for more focus on African populations [7].

A review of studies in sub-Saharan Africa (SSA) found the most common barrier to PCa screening to be a lack of knowledge, followed by perceptions, attitudes, and beliefs [8]. Beliefs that cancer is incurable predominated, while some men had fatalistic views on cancer and would rather not know about it [8], similar to views among rural and urban adults in the USA [9]. Some men viewed PCa as sexually transmitted or a consequence of excessive sexual activity, with older men believing their ceased sexual activity exempted them from risk, while fears of screening pain and religious prohibitions further influenced attitudes towards screening [8]. Low levels of education have been associated with low levels of PCa knowledge and the likelihood of screening [10, 11]. Factors enhancing information seeking regarding screening and PCa risk included family and social support, celebrity endorsements, and media with targeted information [12].

In a study in the Limpopo Province of South Africa, 64.1% of the study population exhibited inadequate knowledge about PCa [13]. Many individuals do not undergo necessary screenings, with 96.7% of respondents who had never had a PSA test, although 53.1% expressed willingness to undergo the test [13]. In a separate study in the Gauteng Province of South Africa, 91.7% of men had heard about PCa, with significant associations between knowledge and factors such as age (*p* ≤ 0.001) [14]. Health status was a predictor of awareness, while both age and health status predicted attitudes towards PC among men, especially in Gauteng [14].

In the study involving primary health care providers, 40.0% demonstrated poor practices, which were linked with negative perceptions of prostate cancer [15]. Nurses and community health workers were found to have poor knowledge regarding prostate cancer, with non-participation in prostate cancer-related continuing medical education being linked with poor knowledge (*p* < 0.001) [15]. Most healthcare providers exhibited neutral attitudes (58.6%), and a negative attitude was significantly associated with not participating in prostate cancer-related continuing medical education (*p* = 0.047) [13, 15]. Rural community-based programmes and heightened awareness campaigns are needed to conscientize men about the risk factors, symptoms, diagnosis, and treatment of PC in rural areas of Limpopo. [13].

Previous studies have highlighted the lack of knowledge and the influence of cultural beliefs and attitudes as major barriers to PCa screening in sub-Saharan Africa [16, 17]. Additionally, healthcare provider practices and attitudes, as well as community-level factors such as education and social support, play critical roles in shaping men’s perceptions and behaviors regarding PCa [18, 19]. Given these complexities and grounded in the Health Belief Model, this study aims to investigate the knowledge, attitudes, practices, cultural beliefs, and perceived risk of PCa among men to provide an understanding of the factors influencing PCa awareness and screening behaviors in these communities, thereby informing tailored interventions to improve early detection and reduce mortality rates.

## Methods

### Study Design, Population, and Sample Size

This study was a cross-sectional study conducted among men in the community in August 2023. The study population consisted of men over the age of 40 years, in selected villages under the Mphaphuli tribal authority (Thulamela) and the Niani tribal authority (Musina local municipality). Men residing in Tshikhudini, Tshiulungoma, and Dididi (Thulamela) and in Nkotswi, Bende Mutale, Masisi, and Madimbo (Niani) were recruited to participate in the study. The estimated men in both villages was *N* = 1963. The sample size calculation was done using Epi Info version 7 software, assuming a cross-sectional survey with a population size of 1963, a 50% knowledge score, and 5% acceptable margin of error for 95% confidence intervals. The estimated sample size was 431, which is calculated from the total population of men in the two tribal villages. Following interest in the study by the initial participants, *n* = 431 men took part in the study.

### Data Collection Instrument

The data was collected using a questionnaire, which was developed using the framework of the African Women Awareness of Cancer (AWACAN) tool that was originally developed to measure awareness of breast and cervical cancer amongst women in sub-Saharan Africa (SSA). This tool was validated for use in South African women with a content validity index (CVI) > 78% for most items, test–retest ICC > 0.75 (good), KR-20 > 0.70 (*good internal consistency*), and significant construct validity (*p* < 0.05) [20], and has been adapted for use in this study. The AWACAN tool was modified for PCa and for use in men in the community. This version was then reviewed by a research coordinator (with a background in nursing) at Tshilidzini hospital, and further amendments were made. This tool was piloted amongst the men in the community who did not participate in the main study, after which any adjustments were made.

The questionnaire was written in English and translated into Tshivenda. The questionnaire was divided into seven sections. Section 1 [18 questions], was on information on Socio-demographic data; Sect. 2 [15 questions] an assessment of prostate cancer awareness; Sect. 3 [15 questions], knowledge of risk factors associated with prostate cancer; Sect. 4 [11 questions], an assessment of knowledge of symptoms of prostate cancer; Sect. 5 [5 questions], assessment of health seeking behavior; Sect. 6 [5 questions], assessment of confidence skills and behavior in relation to a prostate cancer sign or symptom; Sect. 7 [12 questions], assessment of barriers to seeking medical help.

### Data Collection

The survey was conducted by fieldworkers who are fluent in English and Tshivenda and are knowledgeable about the area and fluent in Tshivenda, who are already a part of the SAPCS study. They were trained in conducting the surveys. Once the data had been collected, it was translated back to English. Those who agreed to participate were requested to complete a consent form. The researchers went through the consent forms with the participants and then provided clarifications where participants did not understand. The participants were asked to sign the consent form or give verbal consent before completing the questionnaires.

### Data Management

This study was approved by the University of Pretoria Research Ethics Committee (Ethics Number 831/2020).

The research team ensured that all information was kept confidential. Interviewers received appropriate training to ensure uniformity during interviews. The interviewers reviewed the data with one another to ensure its completeness. Double data entry was done daily for 10% of completed questionnaires as soon as the completed questionnaires were received. The two sets of records were compared, and any discrepancies were checked against the original questionnaire and corrected. All the research data and documents referring to the above-mentioned study were stored at the following address: School of Health Systems and Public Health, University of Pretoria. Storage of the above-mentioned data and documents was maintained for a minimum of 15 years from the commencement of the study. The hard copy data and documents were stored in a locked storage area in lockable offices. The electronic data were password protected and only accessible to staff working on the project.

### Data Analysis

Once the data collection was complete, the data from the questionnaires was captured using Qualtrics and exported to STATA version 16.0 for descriptive analysis and multivariate logistic regression.

## Results

### Characteristics of Study Population

The study included 431 men from the Vhembe district in Limpopo Province, South Africa, with a mean age of 51.16 years (standard deviation = 8.47 years). The awareness of prostate cancer was nearly evenly split, with 210 (48.7%) participants having not heard of prostate cancer and 221 (51.3%) having heard of it.

### Sociodemographic Characteristics

In terms of age, participants ranged from 30 to over 60 years old. Among them, 27 (6.3%) were aged 30–40 years, 211 (49.0%) were aged 41–50 years, 124 (28.7%) were aged 51–60 years, and 69 (16.0%) were over 60 years old. Prostate cancer awareness significantly varied with age (*p* < 0.001), with older participants generally more aware. For relationship status, 163 (37.8%) were married, 123 (28.5%) were living with a partner, 76 (17.6%) were single, 36 (8.4%) were separated or divorced, and 28 (6.5%) were widowed. Awareness of prostate cancer was also significantly associated with relationship status (*p* < 0.001), with married participants showing higher awareness. Regarding education level, 8 (1.9%) had no schooling, 78 (18.1%) had incomplete primary education, 84 (19.5%) had incomplete secondary education, 134 (31.1%) had completed secondary education, and 127 (29.5%) had education beyond secondary school. A significant association was found between education level and prostate cancer awareness (*p* = 0.002), with higher education levels correlating with greater awareness (Table 1).

**Table 1:** Sociodemographic characteristics on prostate cancer among men in the Vhembe district in Limpopo Province, South Africa

**Heard of prostate cancer**	**All participants** **(*N*= 431)**	**No** **(*n* = 210)**	**Yes** **(*n* = 221)**	
**Variable**	***N*(%)**	***n*(%)**	***n*(%)**	***p*-value**
**Age**				< 0.001
30–40 years	27 (6.3)	19 (9.1)	8 (3.6)	
41–50 years	211 (49.0)	118 (56.2)	93 (42.1)	
51–60 years	124 (28.7)	48 (22.8)	76 (34.4)	
> 60 years	69 (16.0)	25 (11.9)	44 (19.9)	
**Relationship status**				< 0.001
Married	163 (37.8)	59 (28.1)	104 (47.1)	
Living together with a partner	123 (28.5)	61 (29.1)	62 (28.1)	
Single	76 (17.6)	53 (25.2)	23 (10.4)	
Separated/divorced	36 (8.4)	17 (8.1)	19 (8.6)	
Widowed	28 (6.5)	15 (7.1)	13 (5.9)	
Did not answer	5 (1.2)	5 (2.4)	0 (0)	
**Level of education**				0.002
No schooling	8 (1.9)	3 (1.4)	5 (2.3)	
Primary incomplete	78 (18.1)	42 (20.0)	36 (16.3)	
Secondary incomplete	84 (19.5)	52 (24.8)	32 (14.5)	
Secondary complete	134 (31.1)	69 (32.9)	65 (29.4)	
More than secondary	127	44 (20.9)	83 (37.5)	
**Language**				< 0.001
Tshivenda	355 (82.4)	193 (91.9)	162 (73.3)	
Xitsonga	60 (13.9)	9 (4.3)	51 (23.1)	
Sepedi	2 (0.5)	6 (2.9)	2 (0.9)	
Shona	11 (2.6)	0 (0)	5 (2.3)	
English	3 (0.7)	2 (0.9)	1 (0.4)	
**Employed**				
Did not answer	2 (0.5)	2 (1.0)	0 (0)	0.006
No	169 (39.3)	96 (45.9)	73 (33.0)	
Yes	259 (60.2)	111 (53.1)	148 (67.0)	
**Social grant**				0.051
Did not answer	9 (2.1)	8 (3.8)	1 (0.4)	
No	309 (71.7)	148 (70.5)	161 (72.9)	
Yes	113 (26.2)	54 (25.7)	59 (26.7)	
**Dwelling or housing**				0.136
Brick house/apartment	405 (94.0)	195 (92.9)	210 (95.0)	
Informal dwelling/shack	11 (2.6)	9 (4.3)	2 (0.9)	
Traditional dwelling/hut	11 (2.6)	4 (1.9)	7 (3.2)	
Other	4 (0.9)	2 (0.9)	2 (0.9)	
**Electricity or solar panel**				0.029
Did not answer	4 (0.9)	2 (0.9)	2 (0.9)	
No	16 (3.7)	13 (6.2)	3 (1.4)	
Yes	411 (95.4)	195 (92.9)	216 (97.7)	
**Tap water in the house**				
Did not answer	56 (12.9)	45 (21.4)	11 (5.0)	< 0.001
No	214 (49.7)	118 (56.2)	96 (43.4)	
Yes	161 (37.4)	47 (22.4)	114 (51.6)	
**Tap water in the yard**				
No	59 (13.7)	41 (19.5)	18 (8.1)	0.001
Yes	372 (86.3)	169 (80.5)	203 (91.9)	
**Tap water in the village**				
Did not answer	1 (0.2)	0 (0)	1 (0.5)	0.425
No	161 (37.4)	83 (39.5)	78 (35.3)	
Yes	269 (62.4)	127 (60.5)	142 (64.2)	
**Toilet in the house or yard**				< 0.001
Did not answer	1 (0.2)	1 (0.5)	0 (0)	
No	32 (7.4)	26 (12.4)	6 (2.7)	
Yes	398 (92.3)	183 (87.1)	215 (97.3)	
**Toilet in the community**				< 0.001
Did not answer	64 (14.9)	49 (23.3)	15 (6.8)	
No	104 (24.1)	37 (17.6)	67 (30.3)	
Yes	263 (61.0)	124 (59.1)	139 (62.9)	
**Have a radio**				0.035
Did not answer	5 (1.2)	3 (1.4)	2 (0.9)	
No	65 (15.0)	41 (19.5)	24 (10.9)	
Yes	361 (83.8)	166 (79.1)	195 (88.2)	
**Have a television**				< 0.001
Did not answer	3 (0.7)	3 (1.4)	0 (0)	
No	38 (8.8)	32 (15.2)	6 (2.7)	
Yes	390 (90.5)	175 (83.3)	215 (97.3)	
**Have a mobile phone or computer**				0.002
Did not answer	5 (1.2)	5 (2.4)	0 (0)	
No	38 (8.8)	26 (12.4)	12 (5.4)	
Yes	388 (90.0)	179 (85.2)	209 (94.6)	
**Have a cell phone**				< 0.001
No	39 (9.1)	31 (14.8)	8 (3.6)	
Yes	392 (90.9)	179 (85.2)	213 (96.4)	
**Have internet access**				0.005
Did not answer	12 (2.8)	11 (5.2)	1 (0.5)	
No	98 (22.7)	61 (29.1)	37 (16.7)	
Yes	321 (74.5)	138 (65.7)	183 (82.8)	

### Awareness and Knowledge of Prostate Cancer

The sources of information about prostate cancer varied among participants (Tables 2 and 3). Clinics and hospitals were the most common sources, reported by 67 (15.6%) participants, followed by radio (82, 19.0%), television (30, 6.9%), newspapers (10, 2.3%), church (8, 1.9%), and other sources (25, 5.8%). However, a significant portion (209, 48.5%) did not specify where they had heard about prostate cancer. Participants’ knowledge of risk factors for prostate cancer showed that 194 (45.0%) were aware that being older than 50 years is a risk factor. Additionally, 190 (44.1%) knew that a family history of prostate cancer is a risk factor, 133 (30.8%) were aware of the risk associated with getting STIs, 124 (28.8%) with having too much sex, and 136 (31.6%) with having unprotected sex. Awareness of smoking cigarettes and drinking alcohol as risk factors was 168 (39.0%) and 156 (36.2%), respectively. Only a small number of participants believed that ancestors (32, 7.4%) or evil spirits (47, 10.9%) could cause prostate cancer.

**Table 2:** Prostate cancer awareness among men in Vhembe district, Limpopo, South Africa

Characteristics	Frequency (*N* = 431)	Percentage (100%)
**Where you heard of prostate cancer**		
Church	8	1.9
Clinic/hospital	67	15.6
Newspaper	10	2.3
TV	30	6.9
Radio	82	19.0
Other sources	25	5.8
Not stated	209	48.5
**Family member or friend with prostate cancer**		
No	366	84.9
Yes	65	15.1
**Family member or friend with prostate cancer**		
No	399	92.6
Yes	32	7.4

**Table 3:** Knowledge of risk factors and symptoms for prostate cancer among men in the Vhembe district, Limpopo, South Africa

Characteristics	Frequency (*N* = 431)		Percentage (100%)
**Knowledge on the risk factors for prostate cancer**			
** Being older than 50 years**			
Don’t know	220		51.0
No	17		3.9
Yes	194		45.0
** Having males with prostate cancer history in the family**			
Don’t know	218		50.6
No	23		5.3
Yes	190		44.1
** Getting STI’s**			
Don’t know	233		54.1
No	65		15.1
Yes	133		30.8
** Having too much sex**			
Don’t know	238		55.2
No	69		16.0
Yes	124		28.8
** Having unprotected sex**			
Don’t know	229		53.1
No	66		15.3
Yes	136		31.6
** Smoking cigarette**			
Don’t know	219		50.8
No	44		10.2
Yes	168		39.0
** Drinking alcohol**			
Don’t know	224		52.0
No	51		11.8
Yes	156		36.2
** Ancestors**			
Don’t know	29		6.7
No	370		85.9
Yes	32		7.4
** Evil spirits cause prostate cancer**			
Don’t know	30		7.0
No	354		82.1
Yes	47		10.9
** Bladder does not empty fully**			
Don’t know		149	34.6
No		41	9.5
Yes		241	55.9
** Frequent urination within 2 h**			
Don’t know		142	32.9
No		71	16.5
Yes		218	50.6
** Can’t hold urine**			
Don’t know		147	34.1
No		73	16.9
Yes		211	49.0
** Urine stops and starts during urination**			
Don’t know		146	33.9
No		59	13.7
Yes		226	52.4
** Strain when urinating**			
Don’t know		142	33.0
No		58	13.4
Yes		231	53.6
**Passing urine many times at night**			
Don’t know		163	37.8
No		97	22.5
Yes		171	39.7
** Persistent lower abdominal/back pain**			
Don’t know		158	36.7
No		75	17.4
Yes		198	45.9
** Blood in urine/semen**			
Don’t know		142	33.0
No		52	12.1
Yes		237	54.9
** Painful ejaculation**			
Don’t know		149	34.6
No		68	15.8
Yes		214	49.6
** Erectile dysfunction**			
Don’t know		150	34.8
No		59	13.7
Yes		222	51.5
** Swelling in the legs or pelvic area**			
Don’t know		153	35.5
No		90	20.9
Yes		188	43.6
** Numbness or pain in the hips or legs**			
Don’t know		166	38.5
No		84	19.5
Yes		181	42.0
** Painful bones**			
Don’t know		163	37.8
No		97	22.5
Yes		171	39.7

### Health-Seeking Behavior

When asked about health-seeking behavior, only 7 (1.6%) participants indicated they would ignore symptoms of prostate cancer, while 385 (89.3%) would not, and 39 (9.1%) were unsure. Consulting health professionals showed variability: 103 (24.0%) would visit a traditional healer, 166 (38.5%) would consult a nurse at a clinic or hospital, 89 (20.7%) would see a doctor at a clinic or hospital, and 57 (13.2%) would go to a private GP or doctor (Table 4).

**Table 4:** Confidence, skills, and health-seeking behavior in relation to prostate cancer signs and symptoms among men in the Vhembe District, Limpopo, South Africa

Characteristics	Frequency (*N* = 431)	Percentage (100%)
**Health seeking behavior for prostate cancer**		
** Would you ignore symptoms of prostate cancer**		
Don’t know	39	9.1
No	385	89.3
Yes	7	1.6
** Self-medicate if there are symptoms**		
Don’t know	43	10.0
No	348	80.7
Yes	40	9.3
** Tell someone if there are symptoms**		
Don’t know	38	8.8
No	97	22.5
Yes	296	68.7
** Who would you tell first**		
Family	125	29.0
Friend	31	7.2
Partner	127	29.5
Other	148	34.3
** Who would you consult with first**		
Traditional healer	69	16.0
Nurse at clinic/hospital	166	38.5
Doctor at clinic/hospital	89	20.7
Private GP/doctor	57	13.2
Prophet/faith healer	11	2.6
Other	39	9.0
** Visit a traditional healer if there are symptoms**		
Don’t know	35	8.1
No	292	67.9
Yes	103	24.0
** If you had symptoms, how soon would you visit a traditional healer**		
< 1 week	65	63.7
1 week to < 1 month	22	21.6
1 month to < 3 months	10	9.8
3 months	5	4.9
** If you had symptoms, how soon would you visit a health center**		
Never	73	17.1
< 1 week	236	55.1
1 week < 1 month	87	20.3
1 month < 3 months	13	3.0
3 months	19	4.4
** Confident in symptoms of prostate cancer**		
Don’t know	52	12.1
No	163	37.8
Yes	216	50.1
** Visited healthcare workers for symptoms**		
Don’t know	110	25.5
No	287	66.6
Yes	34	7.9
** Visited traditional healers for symptoms**		
Don’t know	111	25.8
No	291	67.5
Yes	29	6.7
**Reasons do not visit healthcare workers**		
** Fear of the results**		
Don’t know	40	9.3
No	321	74.5
Yes	70	16.2
** Fear of the procedure**		
Don’t know	40	9.4
No	311	72.7
Yes	77	17.9
** Busy to go to healthcare centers**		
Don’t know	40	9.3
No	337	78.2
Yes	54	12.5
** Long time spent at healthcare centers**		
Don’t know	39	9.1
No	284	65.9
Yes	108	25.1
** No money for transport**		
Don’t know	39	9.1
No	318	74.1
Yes	72	16.8
** No confidence in disclosing the problem**		
Don’t know	37	8.6
No	318	73.8
Yes	76	17.6
** Past bad health experience**		
Don’t know	42	9.7
No	295	68.5
Yes	94	21.8
** Feeling embarrassed**		
Don’t know	37	8.6
No	334	77.5
Yes	60	13.9
** Cultural barrier with healthcare workers**		
Don’t know	40	9.3
No	354	82.1
Yes	37	8.6
** No use going to healthcare center**		
Don’t know	42	9.7
No	334	77.5
Yes	55	12.8
** Things that are not discussed in my culture**		
Don’t know	39	9.1
No	319	74.0
Yes	73	16.9

### Factors Associated with Knowledge of Prostate Cancer

Multivariate analysis revealed several significant factors associated with knowledge of prostate cancer (Table 5). Older age groups showed higher awareness, with adjusted odds ratios (aOR) of 2.71 (95% CI [1.04–7.02], *p* = 0.040) for ages 51–60 years, and 3.70 (95% CI [1.30–10.53], *p* = 0.014) for those over 60 years. Language also played a crucial role, with Xitsonga speakers having an aOR of 6.83 (95% CI [3.18–14.67], *p* < 0.001). Having tap water in the house was strongly associated with higher awareness (aOR = 8.65, 95% CI [3.81–19.7], *p* < 0.001), as was owning a cell phone (aOR = 4.47, 95% CI [1.73–11.57], *p* = 0.002).

**Table 5:** Factors associated with knowledge of prostate cancer among men in the Vhembe district, Limpopo, South Africa

	**Univariate**		**Multivariate**	
**Variable**	**OR (95% CI)**	***p* value**	**aOR (95% CI)**	***p* value**
**Age**				
30–40 years	**Ref**	-	-	-
41–50 years	1.87 (0.78–4.47)	0.158	1.64 (0.67–4.01)	0.281
51–60 years	3.76 (1.53–9.26)	**0.004**	2.71 (1.04–7.02)	**0.040**
> 60 years	4.18 (1.60–10.93)	**0.004**	3.70 (1.30–10.53)	**0.014**
**Relationship status**				
Married	**Ref**			
Living together with a partner	0.58 (0.36–0.93)	**0.024**	0.63 (0.39–1.02)	**0.062**
Single	0.25 (0.14–0.44)	** < 0.001**	0.34 (0.18–0.64)	**0.001**
Separated/divorced	0.63 (0.31–1.31)	**0.220**	0.63 (0.30–1.32)	0.219
Widowed	**0**.49 (0.22–1.10)	0.085	0.34 (0.14–0.83)	**0.017**
Did not answer	**1**	-	-	-
**Language**				
Tshivenda	**Ref**	-	-	-
Xitsonga	**6.75 (3.23–14.13)**	** < 0.001**	6.83 (3.18–14.67)	** < 0.001**
Sepedi	1	-	-	
Shona	0.99 (0.29–3.31)	0.991	1.05 (0.29–3.69)	0.945
English	0.59 (0.05–6.62)	0.673	0.73 (0.06–8.47)	0.804
**Tap water in the house**				
Did not answer	**Ref**	**-**	-	-
No	3.33 (1.63–6.78)	**0.001**	3.37 (1.53–7.39)	**0.002**
Yes	**9.92 (4.73–20.83)**	** < 0.001**	8.65 (3.81–19.7)	** < 0.001**
**Tap water in the yard**				
No	**Ref**	-		
Yes	2.74 (1.51–4.94)	**0.001**	1.98 (1.02–3.84)	**0.044**
**Have a cell phone**				
No	**Ref**	**-**		
Yes	4.61 (2.07–10.29)	** < 0.001**	4.47 (1.73–11.57)	**0.002**

**Logistic regression analysis**

• The outcome variable was either the participants heard about prostate cancer or not

• We used manual forward selection to determine factors associated with knowledge of prostate cancer

• Univariate: we used a cut off p-value of 0.25

• Multivariate: we used a cut off p-value of 0.05 to identify factors associated with knowledge of prostate cancer

• Collinearity and goodness of fit was done.

## Discussion

This study provides a comprehensive analysis of the awareness and knowledge of prostate cancer among men in the Vhembe district of Limpopo Province, South Africa. The results underscore the crucial role of sociodemographic factors in shaping health awareness and behaviors, particularly in relation to prostate cancer.

The study revealed significant associations between age, relationship status, and language with prostate cancer awareness. Older participants demonstrated greater awareness, likely due to increased health concerns and interactions with healthcare services as they age [21]. Married individuals also showed higher awareness, which may be attributed to spousal influence and support in health matters [22]. Language played a crucial role, with Xitsonga speakers exhibiting significantly higher awareness compared to speakers of other languages. These findings suggest that tailored health communication strategies that consider linguistic and cultural contexts are essential for effective public health interventions [23].

Education emerged as a significant factor influencing awareness and knowledge of prostate cancer. Participants with higher levels of education were more likely to be aware of prostate cancer and its risk factors. This finding aligns with existing literature, which highlights education as a critical determinant of health literacy [24, 25]. Higher educational attainment typically equips individuals with better access to health information [26] and the capacity to understand and utilize such information effectively [27]. Therefore, educational interventions aimed at increasing prostate cancer awareness should be prioritized, particularly targeting groups with lower educational levels[28].

The study also explored health-seeking behaviors and found that a significant number of participants would consult traditional healers or delay seeking medical advice for prostate cancer symptoms. This preference for traditional healers highlights the need for culturally sensitive health education that integrates traditional beliefs with modern medical practices [29]. Collaborating with traditional healers and community leaders could bridge the gap between traditional and Western health systems [30], encouraging earlier and more appropriate health-seeking behavior [31].

Despite the relatively high awareness of prostate cancer among the study population, knowledge of specific risk factors and symptoms varied. While a significant portion of participants recognized the importance of age and family history as risk factors, many were unaware of other critical risk factors, such as STIs and lifestyle behaviors, similar to a recent study in South Africa [32]. Additionally, knowledge of prostate cancer symptoms was limited, with many participants unable to identify key symptoms such as frequent urination, blood in urine or semen, and erectile dysfunction. This gap in specific knowledge is concerning as it may impede early detection and timely medical intervention [13].

Early detection of prostate cancer significantly improves treatment outcomes and survival rates [5]. Therefore, public health campaigns should not only aim to increase general awareness but also enhance specific knowledge of risk factors and symptoms [14]. Educational programs should incorporate detailed information about the importance of regular screening and the symptoms that warrant medical consultation.

The findings of this study have important implications for public health interventions in the Vhembe district and similar settings. Interventions should focus on the following to improve prostate cancer outcomes: *Educational Campaigns:* Targeting men with lower educational attainment and providing information in local languages to improve health literacy and awareness. *Community Engagement*: Involving community leaders and traditional healers in health education efforts to address cultural barriers and promote early detection. *Screening Programs:* Implementing and promoting accessible prostate cancer screening programs, especially for older men and those with a family history of prostate cancer. *Comprehensive Health Education:* Providing detailed information about prostate cancer risk factors, symptoms, and the importance of early detection through various communication channels.

### Limitations

This study has some limitations. Its cross-sectional design limits causal inference, and self-reported data may be affected by recall or social desirability bias. Cultural beliefs and symptom knowledge were not deeply explored, and health system accessibility was not assessed, which may influence awareness and behavior.

## Conclusion

In conclusion, this study highlights the critical role of education, sociodemographic factors, and culturally sensitive health communication in enhancing prostate cancer awareness and early detection. By addressing these factors through targeted public health interventions, we can improve prostate cancer outcomes and reduce the burden of this disease in the Vhembe district and beyond. Future research should continue to explore the interplay between education, cultural beliefs, and health behaviors to develop more effective strategies for cancer prevention and control.

## Data Availability

The data that support the findings of this study are available on request from the corresponding author.
